# Acute kidney injury in children hospitalized for acute gastroenteritis: prevalence and risk factors

**DOI:** 10.1007/s00467-020-04834-7

**Published:** 2021-01-07

**Authors:** Pierluigi Marzuillo, Maria Baldascino, Stefano Guarino, Silverio Perrotta, Emanuele Miraglia del Giudice, Felice Nunziata

**Affiliations:** 1 Department of Woman, Child and of General and Specialized Surgery, Università della Campania “Luigi Vanvitelli”, Via Luigi De Crecchio 2, 80138 Naples, Italy; 2 Department of Pediatrics, AORN Sant’Anna e San Sebastiano, via Ferdinando Palasciano, 81100 Caserta, Italy

**Keywords:** Children, Acute kidney injury, Acute gastroenteritis, Acidosis, Vomiting, Diarrhea, Risk factors

## Abstract

**Background:**

We aimed to evaluate prevalence of acute kidney injury (AKI) and its risk factors in children hospitalized for acute gastroenteritis (AGE) to identify early predictors of AKI.

**Methods:**

We retrospectively collected clinical and biochemical data of 114 children (57.9% male; mean age 2.9 ± 2.8 years) hospitalized for AGE. AKI was defined according to Kidney Disease/Improving Global Outcomes creatinine criteria. We considered basal serum creatinine as value of creatinine estimated with Hoste (age) equation assuming basal eGFRs were median age-based eGFR normative values for children ≤ 2 years of age, and eGFR 120 mL/min/1.73m^2^ for children > 2 years. Univariate and multivariate logistic regression models were used to explore associations with AKI. We included in multivariate analyses only variables with significant *p* after Bonferroni correction.

**Results:**

AKI was found in 28/114 (24.6%) patients. No patients required hemodialysis, 2 (1.8%) reached AKI stage 3, 2 (1.8%) AKI stage 2, and 24 (21.0%) AKI stage 1. Mean length of stay was 3.6 ± 1.2, 5.0 ± 1.8, and 10.5 ± 5.8 days, for patients with no, mild, and severe AKI (*p* < 0.001), respectively. Duration of symptoms before hospitalization (OR = 2.5; 95% CI = 1.3–5.0; *p* = 0.006), dehydration > 5% (OR = 43.1; 95% CI = 5.4–344.1; *p* = < 0.001), and serum bicarbonate levels (OR = 1.6; 95% CI = 1.2–2.1; *p* = 0.001) were independent predictors of AKI.

**Conclusions:**

About one quarter of patients hospitalized for AGE may suffer from AKI with a longer stay for patients with more severe AKI. Particular attention, however, should be paid to volemia and kidney health of patients with AGE especially in the presence of increased duration of symptoms before hospitalization, dehydration, and lower serum bicarbonate levels.

**Graphical abstract:**

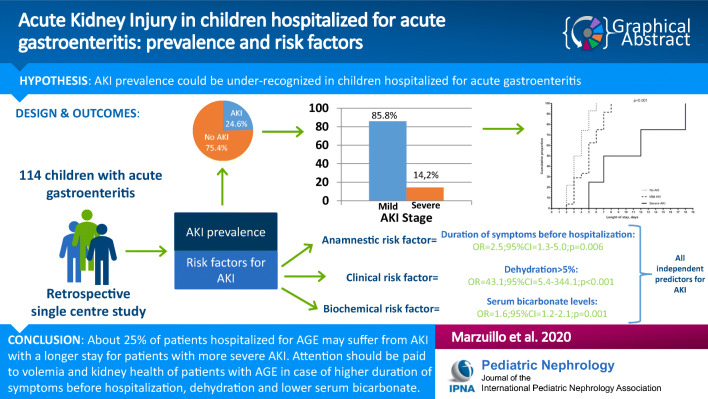

**Supplementary Information:**

The online version contains supplementary material available at 10.1007/s00467-020-04834-7.

## Introduction

Acute gastroenteritis (AGE) is a major cause of morbidity and mortality, with 3–5 billion cases and nearly 2 million deaths occurring each year in children under 5 years of age, mostly in the developing world [1]. The most important and dangerous complications related to AGE are dehydration, metabolic acidosis, and electrolyte disturbances [1].

One of the most common causes of pediatric acute kidney injury (AKI) is kidney hypoperfusion (prerenal AKI) [2]. By this mechanism, AGE could underlie about 12% of the AKI in children [3]. Hospitalized children who develop AKI could experience longer hospital stays and higher mortality in the short-term [4] and increased risk of chronic kidney disease (CKD), hypertension, and proteinuria in the long-term period [5–7].

In Western countries, Bradshaw et al. described that 1% of the patients with infectious diarrheal disease can develop AKI [8]. In this study, however, the incidence of secondary diagnosis of AKI in children hospitalized with a primary diagnosis of diarrheal illness was evaluated searching the entire database on the basis of *International Classification of Diseases, Ninth Revision, Clinical Modification* (ICD-9-CM) diagnosis without having access to clinical and biochemical data. This selection of patients could have determined misclassification of exposure and outcomes, and the lack of access to laboratory data could have underestimated AKI prevalence [8].

We hypothesized that the AKI prevalence could be frequently under-recognized in children hospitalized for AGE, especially for milder forms that could be easily and quickly reverted by rehydration. The detection of milder forms of AKI might allow the interception of patients who could be at risk of later CKD. The fact of having presented even a milder form of AKI, in fact, doubles the risk of CKD [7].

Our study aimed to evaluate the prevalence of AKI and its risk factors in children hospitalized for AGE by means of a direct access to clinical and biochemical data.

## Methods

We retrospectively collected the data of all patients consecutively admitted for AGE to the Pediatric Department of Sant’Anna e San Sebastiano Hospital, Caserta, Italy, from August 1, 2017, to December 31, 2019. This pediatric ward is placed in a general hospital especially devoted to care of adults where pediatricians also manage patients with surgical diseases before referral to a regional Pediatric surgery ward.

**Fig. 1:**
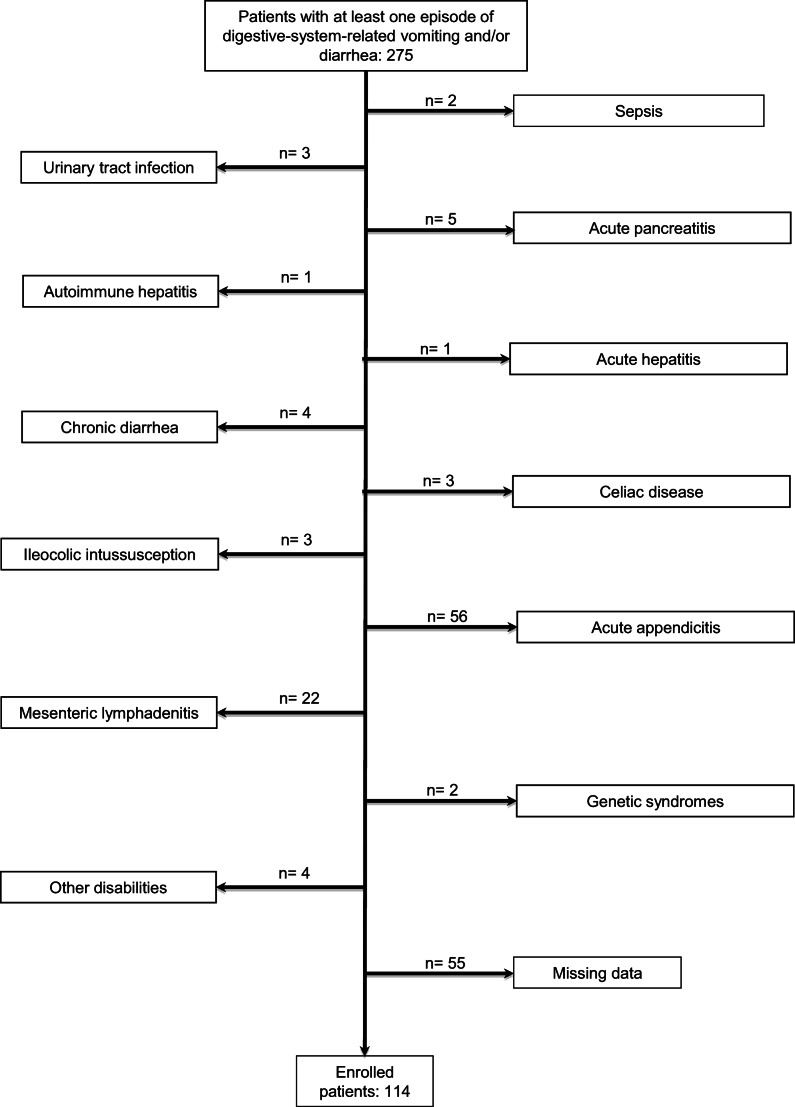
Flow chart describing patients’ enrolment. The 56 patients with acute appendicitis presented at least one episode of vomiting or diarrhea and were excluded because they did not have discharge diagnosis of AGE

Inclusion criteria were the presence of at least one episode of vomiting and/or diarrhea in the last 24 h before hospital admission, discharge primary diagnosis of AGE, and availability of creatinine serum levels both at admission and discharge. We excluded patients in whom we observed complications (i.e., ileocolic intussusception) or other pathologies potentially manifesting or associated with vomiting and/or diarrhea (Fig. 1). We also excluded patients with chronic diarrhea, genetic syndromes, or other disabilities and with missing data regarding serum creatinine values (Fig. 1). None of the patients had previously known nephro-urological diseases.

The study was conducted in accordance with the principles outlined in the Declaration of Helsinki, our Research Ethical Committee approved the study (approval no. 12770/2020), and informed consent was obtained from all the patients before any procedure.

### Data collection

From the digitalized clinical charts, we collected history of known nephro-urological disease, presence of vomiting or diarrhea or both, duration of these symptoms before admission, and birth weight as anamnestic data; indications for hospitalization, age, gender, height, weight, presence of fever, dehydration > 5% or > 10%, blood pressure, heart rate (HR), Glasgow coma scale, and duration of intravenous rehydration as anthropometrical and clinical data; and hematocrit, creatinine, urea, sodium, chloride, potassium, phosphate, calcium, and bicarbonate serum levels and urine dipstick findings as biochemical data at admission. We also recorded the length of stay.

We recorded all the serum creatinine values obtained during hospitalization. In all enrolled patients, we found at least one other creatinine determination in addition to the creatinine at admission and discharge. In 81 out of 114 patients (71%), we had a further creatinine determination after 24 h of stay, in 24 (21%) after 48 h, and in 9 (8%) after 72 h. We recorded the day of hospitalization in which the creatinine levels reached their maximal value. Serum creatinine concentration was measured by IDMS-traceable method. As height was frequently missing, we calculated the estimated glomerular filtration rate (eGFR) by Hoste (age) equation [9].

### Case definition

The primary outcome variable, AKI, was defined by the Kidney Disease/Improving Global Outcomes (KDIGO) according to serum creatinine criteria [10]. We considered as basal serum creatinine value the value of creatinine estimated using previously validated back-calculation methods [11]. As previously described, height-dependent and height-independent basal serum creatinine estimation methods were comparable [11]. Therefore, the Hoste (age) equation was used to back-calculate basal serum creatinine assuming basal eGFRs were the median age-based eGFR normative values for children ≤ 2 years of age [12], and eGFR = 120 mL/min/1.73 m^2^ for children > 2 years [13]. No AKI was defined as occurring if all serum creatinine values were < 1.5 times the basal creatinine, stage 1 AKI if a creatinine value was 1.5 to < 2, stage 2 if 2 to < 3, and stage 3 if ≥ 3 times the basal creatinine [10]. For our analyses, stage 1 AKI was defined as mild AKI and Stage 2 + 3 were defined as severe AKI.

We did not consider the KDIGO urine output criteria because urinary output measurement was lacking.

### Other definitions

Dehydration was classified as < 5%, 5–10%, and > 10% of fluid deficit on the basis of retrospective evaluation of clinical conditions reported in clinical charts and according to the World Health Organization definition [14]. This definition evaluates the dehydration degree on the basis of some clinical parameters including general appearance (ranging from well to lethargic), eyes (normal or sunken), thirst (not thirsty, thirsty, drinks poorly, or not able to drink), and skin turgor (skin goes back quickly, slowly, or very slowly). A patient with no dehydration (< 5% of fluid deficit) should be in well general condition, with normal eyes, not thirsty, and with skin that goes back quickly. A patient with 2 signs from among irritability, sunken eyes, thirsty, and skin turgor that goes back slowly, is moderately dehydrated (5–10% of fluid loss). A patient with 2 signs from among unconscious or lethargic, sunken eyes, drinks poorly or not able to drink, and skin turgor that goes back very slowly, is severely dehydrated (> 10% of fluid loss).

The HR was compared with percentiles for age and body temperature [15]. Increased HR was defined by HR > 2 standard deviation score (SDS).

Impairment of consciousness was defined as present or absent. We defined the impairment of consciousness as present in case of Glasgow coma scale < 15.

Normal values of serum phosphate levels were evaluated on the basis of age in accordance with Clayton et al. [16].

Proteinuria was defined by values ≥ 15 mg/dL at urinary dipstick. We decided to include this low value of proteinuria to not miss patients with low molecular weight proteinuria possibly linked to AKI-related tubular injury.

### Post hoc power calculation

On the basis of AKI prevalence of 1% in children hospitalized with infective diarrheal illness in the USA [8], considering the prevalence of AKI of 24.6% in our population of 114 subjects, the calculated post hoc power, with an alpha of 0.05, was 100%.

### Statistical analysis

*p* values < 0.05 were considered statistically significant. Differences for continuous variables were analyzed with the independent-sample *t* test for normally distributed variables and with the Mann-Whitney test in case of non-normality. Qualitative variables were compared using the chi-squared test. The length of stay was studied by survival analysis according to the Kaplan-Meier method. The day of admission was considered the starting point, while the end point was the date of discharge. Kaplan-Meier curves were compared by log-rank test.

We used the Spearman test to evaluate the correlation between birth weight and both duration of symptoms before admission and length of stay.

Logistic regression models were used with the aim of exploring associations with AKI. We added into the univariate logistic regression analyses parameters that demonstrated association (*p* < 0.05) with AKI after comparison of characteristics of the patients with and without AKI (Table 1). These parameters were grouped in anamnestic, clinical, and biochemical risk factors for AKI in order to give useful information and possible red flags for each of the usual steps of patient evaluation. Among the anamnestic risk factors, duration of symptoms before hospitalization (linear) and birth weight (linear) were included; among clinical risk factors, dehydration > 5% (yes/no), refill > 2 s (yes/no), HR > 2 SDS (yes/no), and Glasgow coma scale < 15 (yes/no) were included; among biochemical risk factors, serum bicarbonates, urea, sodium and phosphorus levels (linear), and hematocrit > 45% (yes/no) were included. In the univariate analysis, we did not include (i) duration of intravenous rehydration and length of stay because these were only a posteriori available data, (ii) dehydration > 10% because clinically strictly linked to dehydration > 5%, (iii) proteinuria because the presence of proteinuria could be a false positive finding due to dehydration. Univariate logistic regression analyses were performed to identify candidate variables to include in the multivariate analysis. We included in the multivariate analysis only the variables with significant *p* after Bonferroni correction at univariate analysis: *p* was considered significant after Bonferroni correction if < 0.025 for anamnestic risk factors, < 0.012 for clinical risk factors, and < 0.01 for biochemical risk factors. In the multivariate analysis, we considered significant only the variables with *p* under the cut-off identified at Bonferroni correction.

**Table 1:** Clinical and laboratory characteristics of all enrolled patients, and of the patients with and without AKI

	All patients *n* = 114	AKI (no) *n* = 86	AKI (yes) *n* = 28	*p*
Age, year	2.9 ± 2.8	3.0 ± 2.7	3.5 ± 2.2	0.23
Male gender, n (%)	66 (57.9)	51 (59.3)	15 (53.6)	0.59
Birth weight, kg	3.1 ± 0.5	3.0 ± 0.5	3.4 ± 0.4	0.001
Weight, percentiles	45.3 ± 30.3	45.1 ± 30.3	40.9 ± 28.6	0.54
Duration of symptoms before admission, days	2.1 ± 0.9	1.9 ± 0.8	2.6 ± 0.99	< 0.001
Presence of fever, n (%)	36 (32)	26 (30.2)	10 (35.7)	0.58
Presence of vomiting, n (%)	97 (85.1)	73 (84.9)	24 (85.7)	0.9
Presence of diarrhea, n (%)	88 (77.2)	63 (73.2)	25 (89.2)	0.08
Presence of vomiting + diarrhea, n (%)	71 (62.3)	50 (58.1)	21 (75)	0.11
Refill > 2 s, n (%)	18 (15.8)	9 (10.5)	9 (32.1)	0.006
HR, beats/min	115.7 ± 12.9	115.5 ± 13.4	116.6 ± 11.5	0.68
HR > 2SDS for age, n (%)	23 (20.2)	12 (13.9)	11 (39.3)	0.004
Glasgow coma scale < 15, n (%)	12 (10.5)	2 (2.3)	10 (35.7)	< 0.001
Dehydration > 5%, n (%)	60 (52.6)	33 (38.4)	27 (96.4)	< 0.001
Dehydration > 10%, n (%)	9 (7.9)	2 (2.3)	7 (25.0)	< 0.001
Duration of intravenous rehydration, h	30.4 ± 28.4	25.2 ± 24.6	46.4 ± 32.4	0.001
Serum bicarbonates levels^a^, mEq/L	19.3 ± 4.2	20.7 ± 3.4	15.2 ± 3.5	< 0.001
eGFR at creatinine peak, mL/min/1.73 m^2^	91.8 ± 28.3	100.6 ± 25.5	64.6 ± 17.2	< 0.001
Urea serum level, mg/dL	13.7 ± 7.4	12.5 ± 4.5	17.0 ± 12.1	0.004
Proteinuria^b^, n (%)	41 (43.2)	17 (25.4)	24 (85.7)	< 0.001
Urinary specific gravity^b^	1019.4 ± 9.2	1019.2 ± 9.3	1019.8 ± 8.9	0.76
Serum Na level, mEq/L	135.7 ± 4.6	136.3 ± 4.4	133.7 ± 6.8	0.009
Serum chloride level, mEq/L	98.7 ± 5.2	99.1 ± 4.43	97.7 ± 6.9	0.21
Serum potassium level, mEq/L	4.4 ± 0.6	4.3 ± 0.57	4.4 ± 0.60	0.68
Serum phosphate level, mg/dL	4.3 ± 1.1	4.6 ± 0.85	3.5 ± 1.1	< 0.001
Serum phosphate level < 2SDS for age, n (%)	17	3 (3.5)	14 (50.0)	< 0.001
Serum calcium level, mg/dL	10.0 ± 0.6	10.1 ± 0.65	9.8 ± 0.55	0.08
Hematocrit > 45%, n (%)	10 (8.8)	4 (4.6)	6 (21.4)	0.006
Length of stay, days	4.1 ± 2.1	3.6 ± 1.2	5.8 ± 3.2	< 0.001

^a^This data was available for 67 patients

^b^This data was available for 95 patients

For normally distributed variables, mean ± SDS are shown, while for non-parametric ones median and lower and upper quartiles are shown

*AKI*, acute kidney injury; *eGFR*, estimated glomerular filtration rate; *HR*, heart rate; *SDS*, standard deviation score

The Stat-Graph XVII software for Windows was used for all statistical analyses with the exception of logistic regression models made with SPSS 25 software for Windows.

## Results

### General characteristics

One hundred fourteen patients (57.9% of male gender) with mean age 2.9 ± 2.8 years (age range: 1 month–13.5 years of age) met inclusion criteria (Fig. 1). In 47 out of 114 (41.2%) patients, the etiology was identified. Thirty-three (29%) patients presented rotavirus infection, 8 (7%) minor salmonellosis, 6 (5.2%) other infectious AGE, and 67 (58.7%) undefined but very likely infectious AGE.

In addition to vomiting and/or diarrhea, indications for hospitalization were poor general clinical condition (*n* = 29), fever (*n* = 36), blood emission with vomiting or diarrhea (*n* = 3), refusal to drink reduced osmolarity oral rehydration solutions (*n* = 26), and poor parental compliance to medical prescriptions (*n* = 20).

The general characteristics of the enrolled patients are shown in Table 1.

All patients needing intravenous rehydration underwent normal saline infusion. The other patients took reduced osmolarity oral rehydration solutions (50/60 mmol/L Na) [17].

Only two patients took one dose of ibuprofen and one patient took acyclovir before hospitalization without having shown AKI. None of the other patients took nephrotoxic drugs before or during hospitalization. In case of fever-related distress, during hospitalization the 36 patients with fever took only paracetamol.

**Fig. 2:**
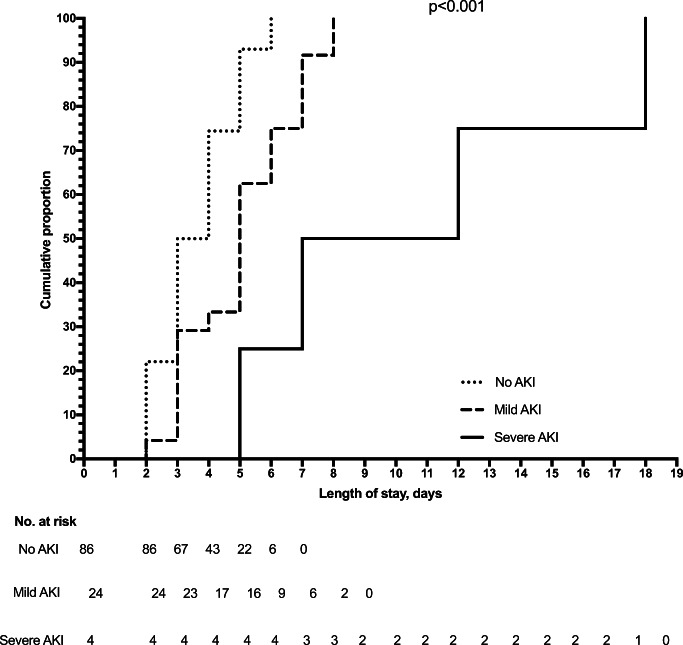
Length of stay evaluated by Kaplan-Meier analysis. The cumulative proportion of discharge of patients without AKI was 22.1% at 2 days, 50% at 3 days, 74.4% at 4 days, 93.0% at 5 days, and 100% at 6 days. For the patients with mild AKI, the cumulative proportion of discharge was 4.1% at 2 days, 29.2% at 3 days, 33.3% at 4 days, 62.5% at 5 days, 75.0% at 6 days, 91.7% at 7 days, and 100% at 8 days. For the patients with severe AKI, the cumulative proportion of discharge was 0% until 4 days, 25% at 4 days, 50% at 7 days, and 100% at 18 days. Log-rank test comparing the three Kaplan-Meier curves showed a global *p* < 0.001 (no vs. mild AKI, *p* < 0.001; mild vs. severe AKI, *p* = 0.01; no vs. severe AKI, *p* < 0.001)

Out of 114 patients, AKI was found in 28 (24.6%). No patient required hemodialysis, 2 (1.8%) patients reached AKI stage 3, 2 (1.8%) AKI stage 2, and 24 (21.0%) reached AKI stage 1. Among the 28 patients with AKI, 19 (67.9%) presented maximum stage at admission, 7 (25%) at 1 day, and 2 (7.1%) at 2 days of hospitalization. None of the enrolled patients developed AKI during hospitalization for causes different from AGE. The mean length of stay was 3.6 ± 1.2 days, 5.0 ± 1.8 days, and 10.5 ± 5.8 days, for patients without AKI, mild AKI, and severe AKI (*p* < 0.001), respectively. These findings were also confirmed by the Kaplan-Meier analysis that showed the shortest time to discharge for patients with no AKI, the longest time for patients with severe AKI, and intermediate time for patients with mild AKI (Fig. 2).

The AKI prevalence was 33.3% (11 out of 33) among patients with rotavirus infection, 37.5% (3 out of 8) among those with minor salmonellosis, 16.7% (1 out of 6) among those with other infectious AGE, and 20.9% (14 out of 67) among those with unidentified but very likely infectious AGE (*p* = 0.45).

Patients with AKI presented higher birth weight, urea levels, duration of symptoms before admission, duration of intravenous rehydration, and length of stay; higher prevalence of refill > 2 s, HR > 2 SDS for age, Glasgow coma scale < 15, dehydration > 5% and > 10%, hematocrit > 45%, serum phosphate levels < 2 SDS and proteinuria; and lower serum bicarbonate, phosphate, and sodium levels, compared with patients without AKI (Table 1).

Patients with proteinuria presented higher urinary specific gravity compared with patients without proteinuria (1022.6 ± 8.7 vs. 1016 ± 8.8; *p* = 0.005).

The Spearman tests showed direct correlations between birth weight and duration of symptoms before admission (*r*^2^ = 0.20 and *p* = 0.04) and birth weight and length of stay (*r*^2^ = 0.37 and *p* < 0.001).

Duration of symptoms before hospitalization and birth weight among anamnestic risk factors; dehydration > 5%, refill > 2 s, HR > 2 SDS for age, and Glasgow coma scale < 15 among clinical risk factors; and serum bicarbonate and phosphorus levels among biochemical risk factors, showed significant associations at the univariate logistic regression analyses and were included in the multivariate analysis (Table 2). At multivariate logistic regression analysis, duration of symptoms before hospitalization among anamnestic risk factors, dehydration > 5% among clinical risk factors, and serum bicarbonate levels among biochemical risk factors were the only significant risk factors (Table 2). A final multivariate analysis including these three parameters showed that all were independent predictors of AKI (Table 2).

**Table 2:** Exploratory analysis of risk factor potentially associated with AKI

	Univariate analysis^f^			Multivariate analysis^f^			Final multivariate analysis^f^		
	OR	95% CI	*p*	OR	95% CI	*p*	OR	95% CI	*p*
Anamnestic risk factors									
Duration of symptoms before hospitalization^a^, days	2.46	1.4/4.2	0.001	2.9	1.6/5.2	0.001	2.5	1.3/5.0	0.006
Birth weight^b^, kg	6.6	1.99/21.8	0.002	3.0	1.05/8.7	0.04	-	-	-
Clinical risk factors									
Dehydration > 5%	43.4	5.6/334.4	< 0.001	28.7	3.6/227.3	0.001	43.1	5.4/344.1	< 0.001
Refill > 2 s	4.1	1.4/11.6	0.009	1.1	0.23/4.9	0.92	-	-	-
Heart rate > 2SDS for age	4.0	1.5/10.6	0.005	1.1	0.29/4.3	0.88	-	-	-
Glasgow coma scale < 15	23.3	4.7/115.7	< 0.001	8.3	1.2/57.4	0.03	-	-	-
Biochemical risk factors									
Serum bicarbonate level^c^, mEq/L	1.6	1.2/2.1	< 0.001	1.5	1.1/2.1	0.009	1.6	1.2/2.1	0.001
Serum urea level^d^, mg/dL	1.1	1.01/1.2	0.02	-	-	-	-	-	-
Serum phosphorus level^e^, mg/dL	3.7	1.9/6.8	< 0.001	2.8	1.2/6.7	0.01	-	-	-
Hematocrit > 45%	5.6	1.4/21.6	0.01	-	-	-	-	-	-
Serum Na level, mEq/L	1.2	1.04/1.3	0.01	-	-	-	-	-	-

^a^1-day increase in symptom duration

^b^1-kg increase in birth weight

^c^1 mEq/L decrease in bicarbonate levels

^d^1 mg/dL increase in urea levels

^e^1 mg/dL decrease in phosphorus levels

^f^The variables with significant *p* after Bonferroni correction were included in the multivariate analysis. The *p* was considered significant after Bonferroni correction if < 0.025 for anamnestic risk factors, < 0.012 for clinical risk factors, and < 0.01 for biochemical risk factors. At multivariate analysis, we considered significant only the variables with *p* under the cut-off identified at Bonferroni correction and only these variables have been included in the final multivariate analysis

*AKI*, acute kidney injury; *SDS*, standard deviation score

## Discussion

This is the first study based on retrospective inspection of the clinical charts investigating AKI prevalence and its risk factors in children hospitalized for AGE in Western countries. The only study available on this topic in Western countries is from Bradshaw et al., who found that only 1% of children with infectious diarrhea developed AKI [8]. This appears very different from the prevalence of 24.6% of our study. Considering that the AKI detected by ICD-9-CM codes is likely to be the severe form, if we compare the prevalence found by Bradshaw et al. [8] with prevalence of severe AKI (3.5%) or stage 3 AKI (1.8%) in our study, the difference is very small. This small difference, however, could still be explained by the different research designs. On the other hand, in the setting of the developing world (defined according to the International Monetary Fund developing countries list [18]), Moghtaderi et al. [19] showed an AKI prevalence in children hospitalized for AGE of 78.6%. The difference with our results could be explained by the different availability of access to medical care in developing countries [20] with subsequent more severe dehydration in the latter patients.

Community-acquired AKI originates due to causative factors acquired in the communities in which patients live [21]. All of our patients presented with AKI as a result of community-acquired AGE. Among the 28 patients with AKI, 26 presented their creatinine peak within 1 day from admission and only in 2 patients within 2 days. Therefore, all our patients met the definition of community-acquired AKI [21]. Moreover, none of them presented AKI later during hospitalization for causes different from AGE.

The risk factors for AKI in children hospitalized for AGE are largely unexplored. Bradshaw et al. identified CKD, hypertension and other kidney and genitourinary chronic conditions as risk factors for AKI in children with infectious diarrheal illnesses, but they did not examine other clinical and biochemical risk factors for AKI [8]. In the present study, we found that duration of symptoms before hospitalization as anamnestic, dehydration > 5% as clinical, and serum bicarbonate levels as biochemical parameters were significant and independent predictors for AKI in children hospitalized for AGE.

An unexpected finding at univariate analysis for AKI risk factors was the increased OR of 6.6 for each increase of 1 kg in birth weight. In fact, it is largely accepted that lower birth weights are associated with reduced nephron mass and increased risk of kidney injury [22]. However, the Spearman tests showed direct correlations between birth weight and duration of symptoms before admission. Possibly, parents of children with low birth weight pay more attention to any acute event of their children with increased proneness to access medical care. Accordingly, we found that with increasing the birth weight the length of stay increased. This suggests that children with lower birth weight showed better general conditions with subsequent shorter length of stay.

Interestingly, the infectious agents were not able to influence the prevalence of AKI. This implies that the effect of vomiting, diarrhea, and subsequent dehydration is the main mechanism underlying AKI with no effect of the AGE etiology.

The prevalence of proteinuria was particularly high (43.2%) in our population. An explanation could be the high urinary specific gravity in dehydrated children. In fact, in our cohort, the patients with proteinuria presented higher urinary specific gravity than those without proteinuria. Therefore, a significant number of patients could have presented a falsely positive proteinuria. For this reason proteinuria does not represent a reliable marker in our retrospective cohort and we did not include this variable in the logistic regression analyses.

The length of stay was shorter for patents with no AKI, intermediate for patients with mild AKI, and longer for patients with severe AKI (Fig. 2). However, from a practical point of view, the difference in length of stay between patients without AKI and with mild AKI was small. This indicates that mild forms of AKI easily and quickly revert. Therefore, in the short-term, mild AKI does not carry a major risk of acute complications and as such its identification may not be relevant. In the long-term, however, the identification of mild forms of AKI could be relevant to schedule a proper follow-up for these patients with periodic evaluation of kidney function, proteinuria, and blood pressure [7]. Of note, none of the patients having shown AKI during AGE in our retrospective analysis underwent nephrological follow-up for this episode, which confirms our hypothesis that AKI could be often overlooked.

Moreover, the parents of these patients should be carefully trained in order to avoid further AKI episodes due to AGE in their child. In fact, in case of repeated AKI, a further increased risk of CKD is possible in adulthood [23].

Some limitations of our study are the retrospective design with lack of a real basal serum creatinine (before hospitalization), the single-center experience, and the limited number of patients. Despite the limited sample size, however, the post hoc power of our study is acceptable. Another limitation was that it was not possible to use the KDIGO urinary output criteria, which could have determined underestimation of AKI prevalence.

In conclusion, we found that—in Western countries—about 25% of patients hospitalized for AGE may suffer from AKI with a longer stay for those with more severe AKI. Main risk factors for AKI in children hospitalized for AGE are represented by duration of symptoms before hospitalization, dehydration > 5%, and serum bicarbonate levels. In accordance with the current guidelines for AGE in childhood [17], oral rehydration should always be preferred and sustained. Particular attention, however, should be paid to volemia and kidney health of these patients, especially in children with longer duration of symptoms before hospitalization, dehydration, and lower serum bicarbonate levels. For a future research perspective, it could be interesting to evaluate the utility of a short intravenous rehydration course as a prophylactic measure against AKI development.

## Supplementary Information

ESM 1 (PPTX 116 kb)

## Data Availability

The dataset generated during and/or analyzed during the current study is available from the corresponding author on reasonable request.
