# Supplementary material for: Acute kidney injury in children hospitalized for acute gastroenteritis: prevalence and risk factors
Source: Pediatr Nephrol. 2021 Jan 7;36(6):1627–35. doi: 10.1007/s00467-020-04834-7 (PMC8084840; doi:10.1007/s00467-020-04834-7)
Supplement: Supplementary file 1 — (PPTX 116 kb) [file 467_2020_4834_MOESM1_ESM.pptx]

## Slide 1
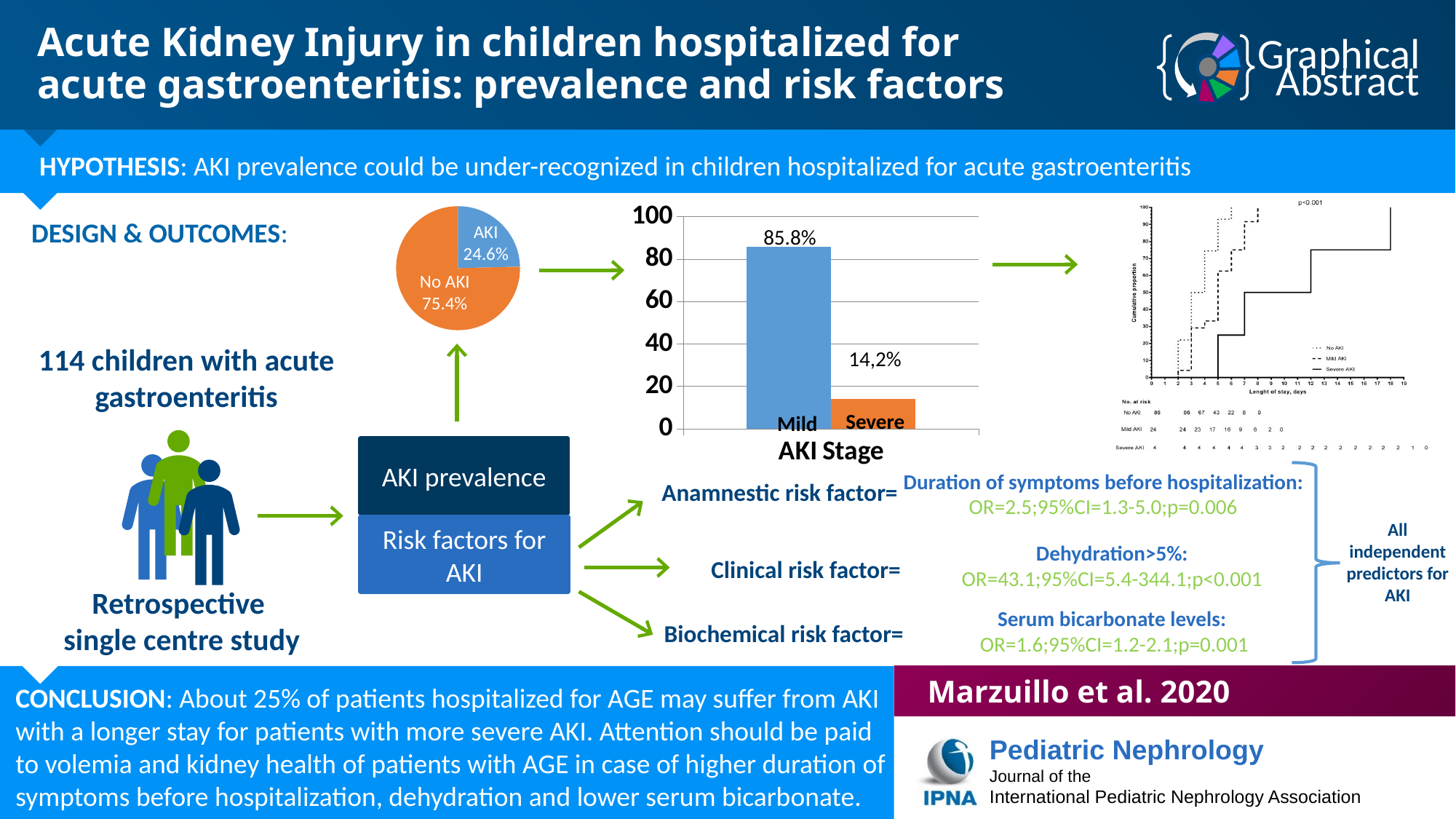

Acute Kidney Injury in children hospitalized for acute gastroenteritis: prevalence and risk factors
HYPOTHESIS: AKI prevalence could be under-recognized in children hospitalized for acute gastroenteritis
### Chart
| Category | AKI prevalence |
|---|---|
| AKI | 24.6 |
| No AKI | 75.4 |AKI
24.6%
No AKI
75.4%
### Chart
| Category | Mild | Severe |
|---|---|---|
| AKI Stage | 85.8 | 14.2 |DESIGN & OUTCOMES:
### Chart
| Category |
|---|85.8%
114 children with acute gastroenteritis
14,2%
Severe
Mild
AKI prevalence
Risk factors for AKI
Duration of symptoms before hospitalization:
OR=2.5;95%CI=1.3-5.0;p=0.006
Anamnestic risk factor=
All independent predictors for AKI
Dehydration>5%: OR=43.1;95%CI=5.4-344.1;p<0.001
Clinical risk factor=
Retrospective
single centre study
Serum bicarbonate levels:
OR=1.6;95%CI=1.2-2.1;p=0.001
Biochemical risk factor=
Marzuillo et al. 2020
CONCLUSION: About 25% of patients hospitalized for AGE may suffer from AKI with a longer stay for patients with more severe AKI. Attention should be paid to volemia and kidney health of patients with AGE in case of higher duration of symptoms before hospitalization, dehydration and lower serum bicarbonate.
